# 12 Monitoring Monocyte Anisocytosis Changes as a Burn Injury Recovery Marker: Insights from a Clinical Study

**DOI:** 10.1093/jbcr/irae036.012

**Published:** 2024-04-17

**Authors:** Saeed Nazemidashtarjandi, Matthew D Supple, Brittany Boribong, Nikki Rosado, Nezihi Murat Karabacak, Lael Yonker, Martin Yarmush, Jeremy Goverman, Daniel Irimia

**Affiliations:** Harvard Medical School, Boston, Massachusetts; Massachusetts General Hospital, North Reading, Massachusetts; Massachusetts General Hospital, Cambridge, Massachusetts; Shriners Hospital for Children, South Boston, Massachusetts; Harvard Medical School, Boston, Massachusetts; Massachusetts General Hospital, Boston, Massachusetts; Shriners Burns Hospital; Harvard Medical School, Boston, Massachusetts; Massachusetts General Hospital, North Reading, Massachusetts; Massachusetts General Hospital, Cambridge, Massachusetts; Shriners Hospital for Children, South Boston, Massachusetts; Harvard Medical School, Boston, Massachusetts; Massachusetts General Hospital, Boston, Massachusetts; Shriners Burns Hospital; Harvard Medical School, Boston, Massachusetts; Massachusetts General Hospital, North Reading, Massachusetts; Massachusetts General Hospital, Cambridge, Massachusetts; Shriners Hospital for Children, South Boston, Massachusetts; Harvard Medical School, Boston, Massachusetts; Massachusetts General Hospital, Boston, Massachusetts; Shriners Burns Hospital; Harvard Medical School, Boston, Massachusetts; Massachusetts General Hospital, North Reading, Massachusetts; Massachusetts General Hospital, Cambridge, Massachusetts; Shriners Hospital for Children, South Boston, Massachusetts; Harvard Medical School, Boston, Massachusetts; Massachusetts General Hospital, Boston, Massachusetts; Shriners Burns Hospital; Harvard Medical School, Boston, Massachusetts; Massachusetts General Hospital, North Reading, Massachusetts; Massachusetts General Hospital, Cambridge, Massachusetts; Shriners Hospital for Children, South Boston, Massachusetts; Harvard Medical School, Boston, Massachusetts; Massachusetts General Hospital, Boston, Massachusetts; Shriners Burns Hospital; Harvard Medical School, Boston, Massachusetts; Massachusetts General Hospital, North Reading, Massachusetts; Massachusetts General Hospital, Cambridge, Massachusetts; Shriners Hospital for Children, South Boston, Massachusetts; Harvard Medical School, Boston, Massachusetts; Massachusetts General Hospital, Boston, Massachusetts; Shriners Burns Hospital; Harvard Medical School, Boston, Massachusetts; Massachusetts General Hospital, North Reading, Massachusetts; Massachusetts General Hospital, Cambridge, Massachusetts; Shriners Hospital for Children, South Boston, Massachusetts; Harvard Medical School, Boston, Massachusetts; Massachusetts General Hospital, Boston, Massachusetts; Shriners Burns Hospital; Harvard Medical School, Boston, Massachusetts; Massachusetts General Hospital, North Reading, Massachusetts; Massachusetts General Hospital, Cambridge, Massachusetts; Shriners Hospital for Children, South Boston, Massachusetts; Harvard Medical School, Boston, Massachusetts; Massachusetts General Hospital, Boston, Massachusetts; Shriners Burns Hospital; Harvard Medical School, Boston, Massachusetts; Massachusetts General Hospital, North Reading, Massachusetts; Massachusetts General Hospital, Cambridge, Massachusetts; Shriners Hospital for Children, South Boston, Massachusetts; Harvard Medical School, Boston, Massachusetts; Massachusetts General Hospital, Boston, Massachusetts; Shriners Burns Hospital

## Abstract

**Introduction:**

Severe burn injuries trigger pathological immune responses. However, our ability to monitor these evolving responses is constrained by the intricate confluence of cellular, biochemical, and metabolic alterations that occur concurrently post-injury. Here, we focus on monocytes as integrators of the changes triggered by the burn injury and potential biomarkers for the immune responses after injury.

**Methods:**

We enrolled patients with major burn injuries (15% or more) and collected blood samples twice a week for the duration of the hospital stay. We measured Monocyte Distribution Width (MDW) using a hematology analyzer. We assessed the association between MDW (an FDA-cleared biomarker for sepsis) and pro-inflammatory cytokines levels released in the serum of burn patients. As a reference, we measured the MDW changes in the presence of pro-inflammatory stimuli, e.g., lipopolysaccharide (LPS), as well as lipid mediators of inflammation resolution, e.g., prostaglandin E2 (PGE2), resolvin D1 (RvD1), and resolvin D2 (RvD2).

**Results:**

We enrolled N = 10 patients with major burn injuries, from which we collected and analyzed N = 78 blood samples. We found that the MDW increases during the first week after a major burn injury (MDW = 24.1) above the threshold of healthy individuals of comparable age (MDW = 20.0) and decreases slowly at discharge. In non-surviving patients, MDW increased for ~10 days post-burn injury (MDW=28.7) and remained high until death. We found the strongest correlations between the MDW values and IL-18 cytokine levels in plasma (Two-tailed Student test, P=0.0095, positive Spearman r coefficient=0.9009).

In blood samples from healthy donors, we found that MDW increased upon LPS stimulation (104 EU/mL, MDW= 31.8), at the higher end of values measured in patients. The addition of RvD2 (5 µM) after LPS stimulation decreased MDW significantly (MDW = 20.1, One-way ANOVA, LPS vs. RvD2 +LPS, P = 0.0002), towards values measured in healthy donors.

**Conclusions:**

In patients with major burns, changes in MDW could serve as a biomarker to track inflammation and its resolution throughout the hospitalization. The ability of RvD2 to decrease MDW is novel and deserves further investigation.

**Applicability of Research to Practice:**

MDW values can be useful for monitoring hospitalized burn patients.

